# Impact of Commercial Food Environments on Local Type 2 Diabetes Burden: Cross-Sectional and Ecological Multimodeling Study

**DOI:** 10.2196/70045

**Published:** 2025-09-08

**Authors:** Kurubaran Ganasegeran, Mohd Rizal Abdul Manaf, Lance A Waller, Nazarudin Safian, Muhammad Faid Mohd Rizal, Wye Lee Chiew, Feisul Mustapha, Khairul Nizam Abdul Maulud

**Affiliations:** 1 Department of Public Health Medicine Faculty of Medicine Universiti Kebangsaan Malaysia Kuala Lumpur Malaysia; 2 Clinical Research Centre Seberang Jaya Hospital Ministry of Health Malaysia Seberang Perai, Penang Malaysia; 3 Occupational Safety and Health Unit Seberang Jaya Hospital Ministry of Health Malaysia Seberang Perai, Penang Malaysia; 4 Public Health Unit Seberang Jaya Hospital Ministry of Health Malaysia Seberang Perai, Penang Malaysia; 5 Department of Biostatistics and Bioinformatics Rollins School of Public Health Emory University Atlanta, GA United States; 6 Earth Observation Centre (EOC) Institute of Climate Change Universiti Kebangsaan Malaysia Bangi, Selangor Malaysia; 7 Penang State Health Department Ministry of Health Malaysia Georgetown, Penang Malaysia; 8 Perak State Health Department Ministry of Health Malaysia Ipoh, Perak Malaysia; 9 Department of Civil Engineering Faculty of Engineering & Built Environment Universiti Kebangsaan Malaysia Bangi, Selangor Malaysia

**Keywords:** local eateries, neighborhood, health, diabetes, policies, attributable fraction, township, urbanization, spatial

## Abstract

**Background:**

Neighborhoods resulting from rapid urbanization processes are often saturated with eateries for local communities, potentially increasing exposure to unhealthy foods and creating diabetogenic residential habitats.

**Objective:**

We examined the association between proximity of commercial food outlets to local neighborhood residences and type 2 diabetes (T2D) cases to explore how local T2D rates vary by location and provide policy-driven metrics to monitor food outlet density as a potential control for high local T2D rates.

**Methods:**

This cross-sectional ecological study included 11,354 patients with active T2D aged ≥20 years geocoded using approximate neighborhood residence aggregated to area-level rates and counts by subdistricts (*mukims*) in Penang, northern Malaysia. We used the National Diabetes Registry complemented with data from medical records across 29 primary care clinics throughout the state. Food establishment data were retrieved from the Open Data Portal sourced through the Penang GeoHub, and urbanization indicators were retrieved from MyCensus 2020. We executed point-level proximity- and density-based area-level analysis through multimodel aspatial and spatial regression methods.

**Results:**

Our final hierarchical linear regression revealed that the distance to food complexes, hawker markets, *kopitiams* (a type of coffee shop), 24-7 convenience stores, fast food outlets, and public markets showed statistically significant associations (*P*<.05) with the age and BMI of patients with T2D. In the multiscale geographically weighted regression model, the adjusted *R*^2^ values ranged from 0.15 to 0.62, with lower values observed across the mainland. The multiscale geographically weighted regression model yielded average β coefficients for densities of *kopitiams* (β=0.256), fast food outlets (β=−0.061), 24-7 convenience stores (β=0.028), supermarkets (β=0.122), public markets (β=0.067), and *nasi kandar* (a type of rice dish) restaurants (β=−0.064), urban growth rate (β=0.189), and population density (β=−0.080; t_65.835_≥1.96 in all cases). We established population-attributable fractions suggesting that, if local neighborhoods underwent township restructuring to remove food complexes, hawker markets, or *kopitiams*, an estimated reduction of 0.21%, 0.27%, and 0.09%, respectively, in the risk of T2D cases in Penang would be anticipated. However, if local neighborhoods underwent township restructuring to add hawker complexes, *nasi kandar* restaurants, fast food outlets, 24-7 convenience stores, public markets, or supermarkets, an estimated reduction of between 0.07% and 0.64% in the number of residents with risk of T2D was estimated.

**Conclusions:**

The reported variations provide insights into the associations between high neighborhood T2D rates and the density of a range of food outlets. We observed that these associations varied by place, providing insight into potential monitoring and policy considerations. This work provides evidence for interpretation at the individual and aggregate levels, shifting public health interventions from a generic to a targeted approach and prompting township planners to restructure food outlet accessibility or availability in local neighborhoods and to develop health behavior interventions for local communities for healthy food purchase and consumption.

## Introduction

### Background

Local changes to land use and neighborhoods are structured within ongoing worldwide urbanization changes, impacting access to food, health, and human livelihood across towns, cities, modern villages, and even distant remote areas [1,2]. As societies consolidate in newer environments further removed from food sources, food production, distribution, and accessibility evolve away from local options to more processed, imported, and commercially driven choices [3]. Due to this, and coupled with increasing options for consumption of food with high fat, caloric, or sugar content, societies are changing and assuming dietary transition roles that coincide with lifestyle and behavior changes often in a more sedentary direction and that potentially increase the risk of noncommunicable diseases because of dietary implications [4]. Urban transformations collectively restructure townships to accommodate more connected human habitats, but such transfigurations also accommodate socioeconomic, infrastructural, and socioecological changes supporting the lifestyle dynamics of urban space development coupled with capitalist industrialization processes aimed to catalyze local economic advancement.

Contemporary neighborhoods resulting from ongoing urbanization processes are often saturated with commercial eateries for access by local communities [4]. Urbanization forcibly transforms food systems according to geographical patterns mediated by food supply, food demand, food delivery, and local consumer choices, driving changing market landscapes and interconnectedness between commercial food system actors and consumers [3-5]. Some local neighborhoods are afflicted by social inequities or inequalities and can be even more susceptible to local food commercialization activities that tend to influence peoples’ preferences regarding food purchasing behaviors according to the availability and accessibility of different food outlets [4,5].

As place matters, we posit a local nutritional transition theory from a geographical epistemology perspective collectively nested within neighborhoods’ modernization and urbanization processes, diving across the commercial and sociospatial complexities shifting access to available food choices for local neighborhood consumers [3]. Under these local neighborhood circumstances, it becomes increasingly difficult for local public health organizations and governments to achieve or even maintain sustainable food consumption in line with United Nations Sustainable Development Goal 2; despite food being available and accessible to people to end hunger, it is challenging for community planners to provide access to healthy and nutritious food within these changing foodscapes [5].

Neighborhood food environments are collectively contextualized with respect to how individuals from residential homes or settlements encounter foods and engage in decision-making as to what foods to purchase and consume [6]. The linkages between neighborhood food environments and local burdens of noncommunicable diseases have been widely established in the United States [7-10], South Asia [11], and Australia [12], but to date, none have been established in Malaysia. As measures of availability and accessibility to food outlets in previous work vary significantly according to geographical settings, culture, or resources available [13], this study explored the linkage between food outlet density and proximity within neighborhoods and type 2 diabetes (T2D) rates in the Malaysian setting.

Studies examining correlations involving food environment availability and accessibility across neighborhoods have accumulated over time. Although earlier work has used different metrics (ie, density- or proximity-based measures) for the associations between neighborhood food environments and the local burden of noncommunicable diseases across different settings based on available resources [13], none have explored how the efforts and metrics used to establish connections could cumulatively be used as evidence to motivate and inform targeted interventions and policies related to the restructuring of local food environments across neighborhoods. Despite the implementation of numerous policies and interventions by the Malaysian government to drive healthy food consumption among citizens (eg, the Malaysia sugar tax, Healthier Choice Logo, and Malaysia Dietary Guidelines), the incidence of obesity and T2D has steadily increased over the past decade [14]. These policies were primarily focused on individual-level behavior regarding adopting healthy eating habits but substantially failed to address the changing landscapes of the distal determinants of health, the environment in which we live, and the exposure to food commercialization activities that can nudge individuals to access or purchase ultraprocessed or cheaper food choices offered by commercial actors within reach, in addition to using poor implementation strategies to tackle or control the changing food landscapes [15].

### Objectives

Such circumstances were not due to failure of the strategies implemented but, instead, highlight how those interventions could not be targeted based on priorities due to limited evidence and metrics used to assess and monitor the impacts of the local food environments on residents’ health. To expand this work and establish baseline evidence for local public health and nutrition epidemiologists to use in implementing targeted place-based interventions for T2D control in Penang, our study aimed to aid in data-based crafting of local policies for healthy township restructuring through the following objectives:

1. Determine the proximity of food outlets to the locus of analysis (ie, neighborhood residence) while concurrently yielding associations between proximity to food outlets and age and BMI of patients with T2D by different distance classifications from the locus of analysis
2. Understand how local T2D rates vary by location across subdistricts (*mukims*) in Penang based on urbanization indicators and available food outlet density
3. Establish a policy-driven metric based on population-attributable fractions (PAFs) through an exponentiated algorithmic regression function within *mukims* to estimate how much a particular food outlet density in a town needs to be restructured to control local T2D rates within *mukims*

## Methods

### Study Design and Setting

We designed and implemented a population-based cross-sectional (ie, to determine proximity to food outlets at the individual level to address objective 1) and ecological (ie, to determine T2D rates that vary by location at the aggregate level, as highlighted in objectives 2 and 3) study design that applied aspatial and spatial methodological approaches to determine the relationships among the built environment (specifically, the food environment), urbanization indicators, individual-level demographics, and T2D burden through proximity- and density-based analytics using small-area methods of investigation [16] by leveraging spatial data analysis.

This study was conducted in the northern region of Malaysia within the state of Penang, a United Nations Educational, Scientific, and Cultural Organization heritage site that hosts high numbers of local commercial food enterprises and many tourism activities. The state consists of 83 *mukims* housing approximately 1.77 million people as of 2020 [17].

### Study Population and Geolocation

Data on 12,815 adults with active T2D aged ≥20 years between 2016 and 2020 from 29 primary care clinics (*klinik kesihatan*) throughout Penang by administrative district were retrieved from the state-level National Diabetes Registry (NDR), a subset of the countrywide NDR [18]. These 12,815 active T2D cases were traced individually from each clinic in Penang using patient records to obtain residential address information, *mukim* information, and individual-level covariate data for point pattern analyses.

All T2D cases’ residential address information at the time of diagnosis was geocoded into a coordinate system (longitude and latitude) upon retrieval from the clinic registry. This enhanced temporal accuracy (ie, allowing neighborhood exposures to be more accurately correlated with T2D cases at the time of diagnosis) as neighborhood landscapes and environment change over time in line with urbanization processes and township development. The valid anonymized residential addresses of patients with T2D (in the standard Malaysian format of unit or block, street, neighborhood or village name, and postal code) in Penang underwent geocoding processes to convert locations to the World Geodetic System 84 coordinate system (longitude and latitude). The geocoding process was conducted using the ezGeocode app (ez34) [19], a tool that allows users to geocode addresses in batches to obtain latitude, longitude, and geographical details for each address within a Google spreadsheet.

**Figure 1:**
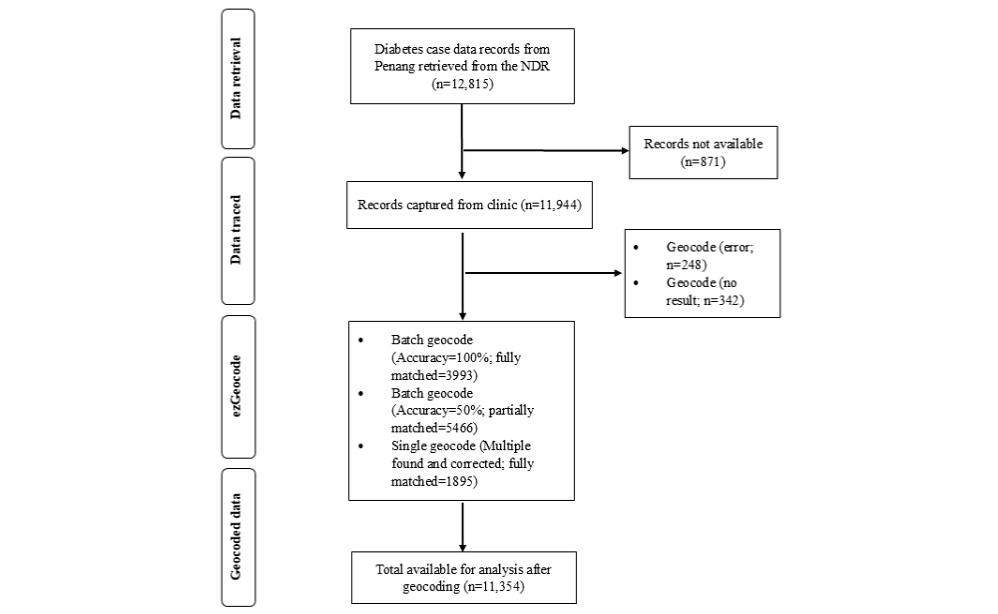
Flowchart of the geocoding process. Geocode (error) means coding returned an error due to an incomplete address or address computed incorrectly. Geocode (no results) means coding returned no results due to limited address information for location detection or no address information. Batch geocoding that returned multiple results was repeated using a single geocode after manual checking for misspelled addresses or street names and plotting on Google Maps via Plus Codes, thus ensuring 100% accuracy of locations via corrected features. NDR: National Diabetes Register.

The process was accomplished by purchasing a quota of 13,000 geocoding locations via the ezGeocode app. The app provided precision information on the batch geocoding results, allowing users to assess the accuracy of the data, with extraction of Plus Codes to provide an additional level of precision via a single geocode [19]. The final sample included 11,354 T2D cases, each of which had a valid residential address successfully geocoded to be compatible within the geographic information system environment for spatial overlay to execute spatial regression analyses. The geocoding process flow and the matched rates of geocoded addresses are available in Figure 1.

### Data Sources

We obtained the official state shapefile (ie, the base map of administrative districts and *mukims*) alongside a digital copyright license for permitted use from the Department of Survey and Mapping Penang (code J72499B; issue date: September 14, 2022) to facilitate spatial overlay for the development of cartographic maps and spatial join of attribute data from various sources.

At the state-wide level, geolocated coordinates of neighborhood food outlets were retrieved from the Penang Open Data Portal sourced through the geographic information system–based Penang GeoHub [20] administrated by the local state government of Penang. A total of 1042 geolocated food outlets were divided into 9 different categories based on outlet type (ie, hawker complexes, food complexes, hawker markets, *kopitiams* (a type of coffee shop), fast food outlets, *nasi kandar* (a type of rice dish) restaurants, 24-7 convenience stores, supermarkets, and public markets); all of these food outlets were classified according to the commercial and economic activities stipulated by the Malaysian Standard Industrial Classification coding scheme for food, beverages, household goods, and grocery services [21] (see the complete classifications and harmonization of definitions for the local setting in Penang in Multimedia Appendix 1). Outlets excluded from the analysis included cafeterias and canteens for childcare and educational institutions, bars, taverns, hotels, caterers, delivery-only outlets, dessert shops, theaters, cinemas, hospitals, bistros, clubs, motels, lodges, campgrounds, suppliers of alcohol, and refreshment rooms as these services were not commonly present within the local neighborhoods [22].

Urban population growth rates, defined as urban population shifts between the years of 2010 and 2020 by *mukim* in Penang, were based on MyCensus 2020 data [23]. Population density, defined as the number of inhabitants living in an area per kilometer squared by *mukim*, was based on MyCensus 2020 data [23].

Individual-level data on T2D cases were obtained from the NDR [18]. The NDR is the largest diabetes register in Malaysia, with patients with active T2D captured across 966 primary care clinics at the local level across all states in Malaysia. The NDR is a web-based application systematically capturing patients with active T2D diagnosed by a clinician along with data entered into the system by trained clinic nurses or medical assistants from patient medical records. The registry captures sociodemographic, diagnosis date, complications, and comorbidities of the disease [24]. We acknowledge that data from the NDR rely heavily on documentation by health care providers in the patient case report notes. When data were classified as “unknown” by the registry due to inability to capture them from the clinical documentation [24], they were excluded from the analysis to establish better data accuracy and analysis quality. However, BMI (ie, body weight divided by the square of the height, expressed as kg/m^2^) of patients with T2D and address information were not readily available from the registry. To obtain this information, we referred to the source documentation from the medical records of the diagnosed T2D cases in the clinics. We trained research assistants to visit each clinic after approval from the Penang State Health Department, and with the assistance of a local clinic nurse or medical assistant in charge of managing the NDR in each of the 29 primary care clinics, these research assistants manually traced patient medical records that matched the registration number of T2D cases as captured in the registry. The required individual data (eg, BMI and address information) were extracted manually and tabulated into the master dataset.

### Measures

For point-level food outlet proximity analyses, we used T2D case counts by *mukim* in Penang. For density-based analysis, our main outcome measure was *mukim*-wise T2D crude rates computed from aggregated cases reported on the NDR divided by the total adult population aged ≥20 years by *mukim* in Penang. We log10 transformed T2D crude rates to meet the assumption of normal errors and provide a more symmetrical distribution of the outcome and applied empirical Bayes smoothing to allow for fit of a local spatial regression model that predicts T2D burden varying by space [25]. Food outlet coverage was computed as density per 1000 *mukim* residents.

### Statistical Analyses

#### Proximity-Based Measures

The proximity between each T2D case residential address (ie, the locus of analysis) and food outlets was computed by using the “distance to the nearest hub” feature in the QGIS Bialowieza processing toolbox (version 3.22; QGIS Development Team), an algorithm that computes the distance between point features taken as origin and their closest destination line or polygon feature [13,26,27]. Descriptive statistics, alongside their associated measures of dispersion with minimum and maximum values of the distance, were reported.

Next, we executed locally estimated scatterplot smoothing (LOESS) regression. The LOESS regression is a nonparametric regression technique that uses locally weighted regression to fit a smooth curve through points in a scatterplot (ie, associations between proximity to food outlets in kilometers and age and BMI of T2D cases). If the trend line shows a near smooth line of associations within the weighted scatterplot, the associations could be modeled through conventional multivariate linear regression. The proximity to food environments was analyzed as both a continuous and categorical covariate. The following cutoffs for categories were determined: <0.5 km, 0.5 to <1.0 km, and ≥1.0 km. A minimum cutoff of 0.5 km was first set based on previous evidence that this distance is highly walkable within neighborhoods [28], followed by increments of 500 m for subsequent categories up to ≥1.0 km.

Conventional hierarchical multivariable linear regression analyses using the Enter technique [29] were subsequently executed if the LOESS regression showed a relatively smooth linear pattern of distribution in the correlation between 2 variables (ie, proximity to food outlets and age and BMI of T2D cases). The linear regression analysis took the form of the following equation: *Y* = β_0_ + β_1_*X*_1_ + β_2_*X*_2_ + ... + β_p_*X*_p_, where *Y* is the predicted or expected value of the dependent variable (ie, BMI of T2D cases), *X*_1_ to *X_p_* are *p* distinct covariates (ie, age and different level of proximity to food outlets), β_0_ is the value of *Y* when all covariates (*X*_1_ to *X_p_*) are equal to 0, and β_1_ to β_p_ are estimated regression coefficients for each of the *p* parameters. Each regression coefficient represents the change in *Y* relative to a unit change in the corresponding covariate when all other independent variables are held constant (ie, when the remaining covariates are fixed). The model performance at each step was evaluated by calculating the model base and change statistic parameters. The analysis was conducted using the SPSS software (version 23.0; IBM Corp).

When determining variable selection and fit assessment for models that used conventional regression approaches, covariates were selected based on theoretical and plausible importance. Variables of food outlet proximity were included as these variables have plausible influences on township restructuring for healthy urbanism in preventing T2D. Individual-level factors such as age were included as it was biologically plausible for them to influence T2D. Model fit for linear regression analysis was assessed using *R*^2^ values, and potential multicollinearity between covariates was assessed based on the values of the variance inflation factor exceeding 5. We acknowledge the difficulty of model building for real-life interpretations and acknowledge the dictum of building models with a purpose based on theoretical knowledge that sets the direction of the findings to be adopted for public health policy change [30,31].

#### Density-Based Measures Across Mukims Using Spatial Weights

We applied multiscale geographically weighted regression (MGWR) to explore spatial heterogeneity in the observed associations among neighborhood food outlet density covariates, urbanization indicators, and logged T2D crude rates across space (hereafter simply referred to as T2D rates). The MGWR model yields a set of locally varying regression coefficients that are mapped and geovisualized to document varying spatial associations between neighborhood covariates and T2D rates across *mukims* [32,33]. We initially fit using a log-linear regression (ie, the ordinary least squares model of log-rates) and, subsequently, using the MGWR (which is the log-linear regression), but in this case, we allowed for a smooth variation of the associations (β values) over space.

MGWR is an extension of geographically weighted regression, which is defined by the following equation:




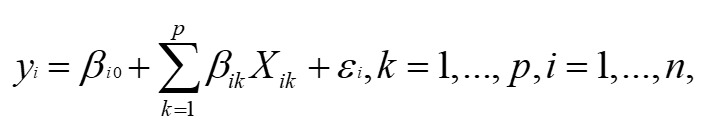




In this equation, *y_i_* is the value of the outcome variable (ie, log10 of T2D rates by *mukim*) for location *i*, β*_i0_* is the intercept coefficient specific to location *i*, *X_ik_* is the value of the *k*th explanatory variable specific to location *i*, β*_ik_* denotes the local regression coefficients for the *k*th explanatory variable specific to location *i*, and ε*_i_* is the random error terms at location *i* assumed to follow an independent normal distribution of zero mean and homogeneous variance [34].

The MGWR allows the scale of all covariates of associations with the main outcome variable to be specific to each covariate. At different spatial locations of the study setting (ie, *mukims* across Penang), these associations may exhibit different impacts on local log-T2D rates. MGWR facilitates spatial variation in estimated associations via a weighting scheme (ie, kernel bandwidth) for each spatial feature using the covariate and main outcome variable of the focal feature areas and their neighbors, with neighboring regions closer to the focal feature area assigned higher weights and larger influence in the local regression model. The geographic scale of correlation is determined via distance-based kernel weights (with either a location-adaptive kernel bandwidth or a simpler fixed bandwidth over space [32]). The kernel weights, defined based on distance between regions (*d_ij_*) and the bandwidth *h*, are given by the following equation [34]:




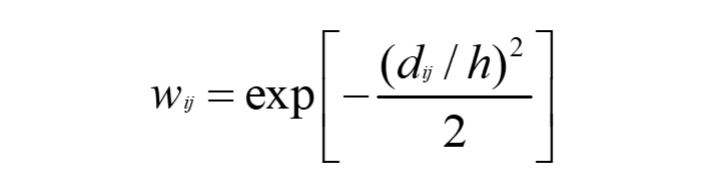




Bandwidths define the effective number of parameters (ENP) that exhibit patterns for interpretations of spatial heterogeneity, where larger ENP values suggest greater spatial heterogeneity. Specifically, ENP values nearing 1 suggest little spatial variation in estimates of associations in local regressions. Covariate-specific adjusted *t* values were obtained [35].

This study applied MGWR modeling to food outlet density features and urbanization indicators at the *mukim* level within the state of Penang, allowing estimated covariate associations to change over time and to operate at different spatial scales or features. As the density of food environment features changes over time at different spatial scales (perhaps because underlying township restructuring and urbanization processes change over time and location as well), understanding local variations in coefficient estimates yielded through MGWR can provide more useful interpretations and insights for local public health interventions and policy planning [36]. In this study, we executed MGWR using adaptive kernels via the MGWR software (version 2.2; Spatial Analysis Research Centre, Arizona State University).

#### Density-Based Measures Within Mukims

We then modeled count outcomes directly by applying a Poisson regression that adjusted for population size by region (ie, *mukims*). A typical Poisson regression is defined by the following equation: *E*(*Y_i_*/*n_i_*) = exp(β_0_ + β_1_ × *X*_1_*_i_* + ... β*_p_* × *X_pi_*).

On the basis of this equation, the law of probability yields the following final Poisson regression equation: *E*(*Y_i_*) = exp[ln(*n_i_*) + β_0_ + β_1_ × *X*_1_*_i_* + ... β*_p_* × *X_pi_*], where β for the log-population value (ln[*n_i_*]) is fixed and equal to 1, defined as the *offset* variable.

The associations between T2D case counts with an offset variable of log-population for *mukims* in Penang and the density of food outlets was explored through Poisson regression modeling executed through generalized linear models using the GENLIN command in the SPSS software (version 23.0).

The Poisson regression analysis yields β coefficients that, when exponentiated, can be interpreted as approximations to the relative risks (ie, rate ratios [RRs]) associated with the outcome. Next, we used model-predicted counts to define the PAFs associated with each covariate to enhance real-life interpretations for proposing public health policy changes. More specifically, the PAF is defined as the proportional reduction of a population being attributed to a disease if a particular risk factor is removed or eliminated. The PAF was calculated after determining the strength of associations between risk exposures and T2D rates through yielded estimates of adjusted RRs (ARRs) and 95% CIs in the Poisson regression analysis using the formula by Miettinen as follows:




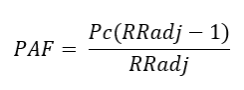




In this formula, *RRadj* is the estimate of the ARR, and *Pc* is the prevalence computed from exposure among the cases [37-39].

### Ethical Considerations

Ethics approval was granted by the ethics committee of the National University of Malaysia; the Ministry of Higher Education Malaysia (JEP-2022-445); and the Medical Research Ethics Committee, Ministry of Health Malaysia (ID-22-01264-EE7; investigator initiated research). The registry data used in this study were deidentified and anonymized to protect patients’ confidentiality, privacy, and anonymity. No information is presented in this paper that would allow for the direct or indirect identification of individuals. The Medical Research Ethics Committee approval covered permissions for secondary data analysis without additional consent. No financial compensation was provided as this study did not involve human participants in primary data collection.

## Results

### Proximity of Local Commercial Food Outlets to the Locus of Analysis

A total of 11,354 people with T2D had access to approximately 1042 food outlets across neighborhoods in Penang. The mean distance to any food outlet accessible to patients with T2D was 0.42 (SD 0.515) km, and the distance ranged from 0 to 5.11 km. The median distance was 0.25 (IQR 0.339) km. Being the highest-distributed food outlet type in Penang, hawker markets (n=175) had the shortest distance of accessibility by local neighborhood residents with a mean of 0.69 (SD 0.642) km, and the distance ranged from 0 to 5.11 km. The median distance to the nearest hawker market was 0.50 (IQR 0.593) km. In contrast, food complexes and supermarkets had the lowest distribution throughout the state (n=43 each), and these outlets were the furthest from local neighborhood residents with a mean distance of 6.58 (SD 7.434) and 2.11 (SD 2.240) km, respectively (Table 1).

**Table 1:** “Distance to the nearest hub” analysis of proximity of food outlets to the neighborhood residences of type 2 diabetes cases (N=11,354).

Outlet type	Outlets, n	Distance (km), mean (SD)	Distance (km), median (IQR)	Range (km)
Any food outlet	1042	0.42 (0.515)	0.25 (0.339)	0-5.11
Hawker complex	116	1.17 (1.168)	0.72 (1.187)	0-7.69
Food complex	43	6.58 (7.434)	3.31 (11.074)	0-33.31
Hawker market	175	0.69 (0.642)	0.50 (0.593)	0-5.11
*Kopitiam*	106	1.87 (1.906)	1.11 (1.580)	0-12.30
*Nasi kandar* restaurant	169	1.03 (1.286)	0.59 (0.828)	0-11.40
Fast food outlet	174	1.63 (1.742)	0.90 (1.473)	0-11.30
24-7 convenience store	159	1.07 (1.341)	0.60 (0.851)	0-10.20
Public market	57	1.32 (1.303)	0.83 (1.396)	0.12-8.80
Supermarket	43	2.11 (2.240)	1.17 (2.155)	0.02-13.06

### Multivariable Linear Regression Analysis on the Associations Between Proximity to Food Outlets and Age and BMI of T2D Cases

The LOESS regression interpolation associations between BMI and food outlet type showed a relatively smooth fit line indicative of linear correlations, and we focus on linear regressions in the following paragraphs (Multimedia Appendices 2-4).

Table 2 shows the results of multivariable linear regression analysis on the associations between proximity to food outlets and age and BMI of T2D cases. A total of 21 covariates accounted for 6.5% of the total variance for the BMI values of T2D cases within neighborhoods in Penang. The base covariates in step 1 accounted for 1.1% of the variance in the BMI of T2D cases within neighborhoods (*F*_6,11,046_ change=21.061), and among those variables, proximity to food complexes of <0.5 km and 0.5 to <1 km (β=−1.130 and −0.756, respectively; *P*<.001) and proximity to hawker markets of <0.5 km and 0.5 to <1 km (β=−0.325 and −0.498, respectively; *P*=.02 and *P*<.001) showed statistically significant negative associations with the BMI of T2D cases. The associations between BMI and proximity to hawker complexes were not statistically significant (*P*>.05; Table 2).

The total variance explained increased slightly by 0.3% after inclusion of *kopitiam*, *nasi kandar* restaurant, and fast food outlet proximity in the second step (*F*_6,11,040_ change=5.007), and the statistically significant variables associated with BMI included proximity to food complexes of <0.5 km and 0.5 to <1 km (β=−1.073 and −0.685, respectively; *P*<.001), proximity to hawker markets of <0.5 km (β=−0.308; *P*=.03) and 0.5 to <1 km (β=−0.481; *P*=.002), proximity to *kopitiams* of <0.5 km (β=0.303; *P*=.049) and 0.5 to <1 km (β=0.502; *P*=.001), and proximity to fast food outlets of <0.5 km and 0.5 to <1 km (β=−0.694 and −0.660, respectively; *P*<.001). Associations between BMI and proximity to hawker complexes and *nasi kandar* restaurants were not statistically significant (*P*>.05; Table 2).

Adding covariates of 24-7 convenience stores, public markets, and supermarkets in the third step contributed minimally to an additional 0.1% of the explained variance in the BMI of T2D cases (*F*_6,11,034_ change=1.382). The statistically significant covariates associated with the BMI of T2D cases were proximity to food complexes of <0.5 km and 0.5 to <1 km (β=−1.041 and −0.649, respectively; *P*<.001), proximity to hawker markets of 0.5 to <1 km (β=−0.444; *P*=.006), proximity to *kopitiams* of <0.5 km (β=0.348; *P*=.04) and 0.5 to <1 km (β=0.560; *P*=.001), proximity to fast food outlets of <0.5 km (β=−0.605; *P*<.001) and 0.5 to <1 km (β=−0.576; *P*=.001), and proximity to 24-7 convenience stores of <0.5 km (β=−0.376; *P*=.03) and 0.5 to <1 km (β=−0.438; *P*=.01). None of the associations between BMI and proximity to hawker complexes; proximity to hawker markets of <0.5 km; and proximity to *nasi kandar* restaurants, public markets, or supermarkets were statistically significant (*P*>.05; Table 2).

Finally, adding age covariates explained an additional 5% of the variance in the BMI of T2D cases (*F*_3,11,031_ change=198.191). The final step in the model found that proximity to food complexes of <0.5 km (β=−0.855; *P*<.001) and 0.5 to <1 km (β=−0.449; *P*=.004); proximity to hawker markets of <0.5 km (β=−0.308; *P*<.04) and 0.5 to <1 km (β=−0.474; *P*=.002); proximity to *kopitiams* of 0.5 to <1 km (β=0.551; *P*=.001); proximity to fast food outlets of <0.5 km (β=−0.457; *P*=.005) and 0.5 to <1 km (β=−0.536; *P*=.001); proximity to 24-7 convenience stores of 0.5 to <1 km (β=−0.348; *P*=.04); proximity to public markets of <0.5 km (β=0.344; *P*=.01); and ages of 20 to 34 years, 35 to 49 years, and 50 to 64 years (β=4.180, 3.221, and 1.386, respectively; *P*<.001) associated with BMI change were statistically significant. Associations between BMI and proximity to hawker complexes, proximity to *kopitiams* of <0.5 km, proximity to *nasi kandar* restaurants, proximity to public markets of 0.5 to <1 km, and proximity to supermarkets were not statistically significant (*P*>.05; Table 2).

**Table 2:** Multivariable linear regression analysis on the associations between proximity to food outlets and age and BMI of type 2 diabetes cases (N=11,047)^a^.

Characteristic	Step 1^b^				Step 2^c^				Step 3^d^				Step 4^e^		
	β (SE)	*P* value	VIF^f^	β (SE)		*P* value	VIF	β (SE)		*P* value	VIF	β (SE)		*P* value	VIF
Hawker complex at <0.5 km	−0.028 (0.131)	.83	1.641	0.115 (0.143)		.42	1.955	0.109 (0.144)		.45	1.974	0.173 (0.140)		.22	1.975
Hawker complex at 0.5 to <1 km	−0.247 (0.147)	.09	1.534	−0.062 (0.156)		.69	1.725	−0.055 (0.157)		.73	1.750	0.015 (0.153)		.92	1.751
Food complex at <0.5 km	−1.130 (0.139)	<.001^g^	1.337	−1.073 (0.140)		<.001^g^	1.375	−1.041 (0.151)		<.001^g^	1.582	−0.855 (0.147)		<.001^g^	1.587
Food complex at 0.5 to <1 km	−0.756 (0.148)	<.001^g^	1.303	−0.685 (0.151)		<.001^g^	1.363	−0.649 (0.158)		<.001^g^	1.492	−0.449 (0.154)		.004^g^	1.496
Hawker market at <0.5 km	−0.325 (0.142)	.02^g^	1.985	−0.308 (0.145)		.03^g^	2.096	−0.280 (0.150)		.06	2.236	−0.308 (0.146)		.04^g^	2.237
Hawker market at 0.5 to <1 km	−0.498 (0.154)	.001^g^	1.842	−0.481 (0.156)		.002^g^	1.894	−0.444 (0.161)		.006^g^	2.001	−0.474 (0.156)		.002^g^	2.001
*Kopitiam* at <0.5 km	—^h^	—	—	0.303 (0.154)		.049^g^	1.710	0.348 (0.166)		.04^g^	1.981	0.264 (0.162)		.10	1.982
*Kopitiam* at 0.5 to <1 km	—	—	—	0.502 (0.154)		.001^g^	1.619	0.560 (0.167)		.001^g^	1.911	0.551 (0.163)		.001^g^	1.911
*Nasi kandar* restaurant at <0.5 km	—	—	—	0.002 (0.153)		.99	2.324	0.068 (0.163)		.68	2.642	−0.026 (0.159)		.87	2.644
*Nasi kandar* restaurant at 0.5 to <1 km	—	—	—	−0.162 (0.160)		.31	1.937	−0.069 (0.171)		.69	2.220	−0.134 (0.167)		.42	2.220
Fast food outlet at <0.5 km	—	—	—	−0.694 (0.154)		<.001^g^	1.900	−0.605 (0.167)		<.001^g^	2.237	−0.457 (0.163)		.005^g^	2.241
Fast food outlet at 0.5 to <1 km	—	—	—	−0.660 (0.154)		<.001^g^	1.803	−0.576 (0.166)		.001^g^	2.094	−0.536 (0.162)		.001^g^	2.095
24-7 convenience store at <0.5 km	—	—	—	—		—	—	−0.376 (0.172)		.03^g^	2.924	−0.289 (0.168)		.09	2.926
24-7 convenience store at 0.5 to <1 km	—	—	—	—		—	—	−0.438 (0.174)		.01^g^	2.387	−0.348 (0.170)		.04^g^	2.389
Public market at <0.5 km	—	—	—	—		—	—	0.208 (0.138)		.13	1.718	0.344 (0.135)		.01^g^	1.722
Public market at 0.5 to <1 km	—	—	—	—		—	—	0.117 (0.148)		.43	1.548	0.187 (0.145)		.20	1.549
Supermarket at <0.5 km	—	—	—	—		—	—	−0.067 (0.168)		.69	1.910	−0.053 (0.164)		.75	1.911
Supermarket at 0.5 to <1 km	—	—	—	—		—	—	−0.031 (0.167)		.85	1.892	0.005 (0.162)		.97	1.893
Age of 20 to 34 y	—	—	—	—		—	—	—		—	—	4.180 (0.293)		<.001^g^	1.098
Age of 35 to 49 y	—	—	—	—		—	—	—		—	—	3.221 (0.144)		<.001^g^	1.493
Age of 50 to 64 y	—	—	—	—		—	—	—		—	—	1.386 (0.120)		<.001^g^	1.512

^a^The reference for the age variable was those aged ≥65 years; the reference for proximity to all food outlets was ≥1 km.

^b^*F*_6,11,046_ change=21.061; *R*^2^=0.011; *R*^2^ change=0.011.

^c^*F*_6,11,040_ change=5.007; *R*^2^=0.014; *R*^2^ change=0.003.

^d^*F*_6,11,034_ change=1.382; *R*^2^=0.015; *R*^2^ change=0.001.

^e^*F*_3,11,031_ change=198.191; *R*^2^=0.065; *R*^2^ change=0.050.

^f^VIF: variance inflation factor.

^g^Statistical significance at *P*<.05.

^h^Not applicable.

### Local Associations Among Neighborhood Food Environments, Urban Indicators, and T2D Rates by *Mukim* in Penang

The MGWR model concluded that variations in local logged T2D rates were positively associated with the density of *kopitiams* per 1000 residents (β=0.256), density of 24-7 convenience stores per 1000 residents (β=0.028), density of supermarkets per 1000 residents (β=0.122), density of public markets per 1000 residents (β=0.067), urban growth rate (β=0.189), and population density (β=0.080). In contrast, the MGWR model found that variations in local logged T2D rates were negatively associated with the density of fast food outlets per 1000 residents (β=−0.061) and the density of *nasi kandar* restaurants per 1000 residents (β=−0.064; t_65.835_≥1.96; Multimedia Appendix 5).

### Variation of Pooled Factors by Location

**Figure 2:**
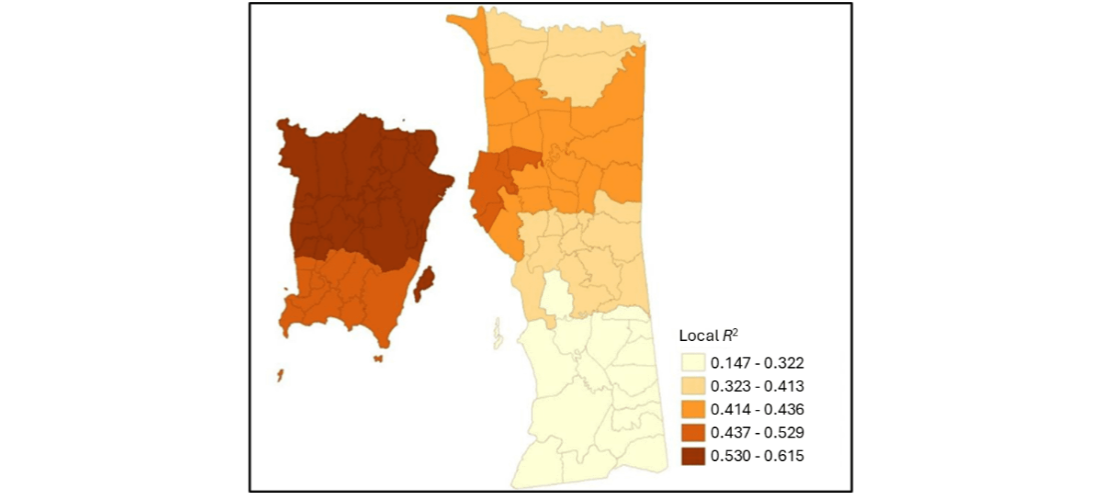
Local *R*^2^ values of pooled covariates.

The local *R*^2^ values from the MGWR model are mapped in Figure 2. The local *R*^2^ values indicate how well the modeled covariates (ie, neighborhood food outlet density and urban indicators) explain the variation in T2D rates in different *mukims* in Penang. Higher *R*^2^ values in certain *mukims* suggest that the model performed well for those locations (ie, the covariates significantly influenced T2D rates in those areas), whereas lower *R*^2^ values suggest that the model explained less of the variance, suggesting that there are other unaccounted for factors that may be influencing T2D rates in those areas. The largest values, ranging from 0.53 to 0.62, were observed across *mukims* in Timur Laut and half of all *mukims* in the Barat Daya district, suggesting that the model has significant predictive power in those areas. Overall, local *R*^2^ values ranged from 0.15 to 0.62 across *mukims* in Penang, but lower values were predominantly observed in mainland Penang, particularly across *mukims* in Seberang Perai Selatan and parts of *mukims* in Seberang Perai Tengah and Seberang Perai Utara.

### Variation of Each Factor That Influenced T2D Rates by Location

**Figure 3:**
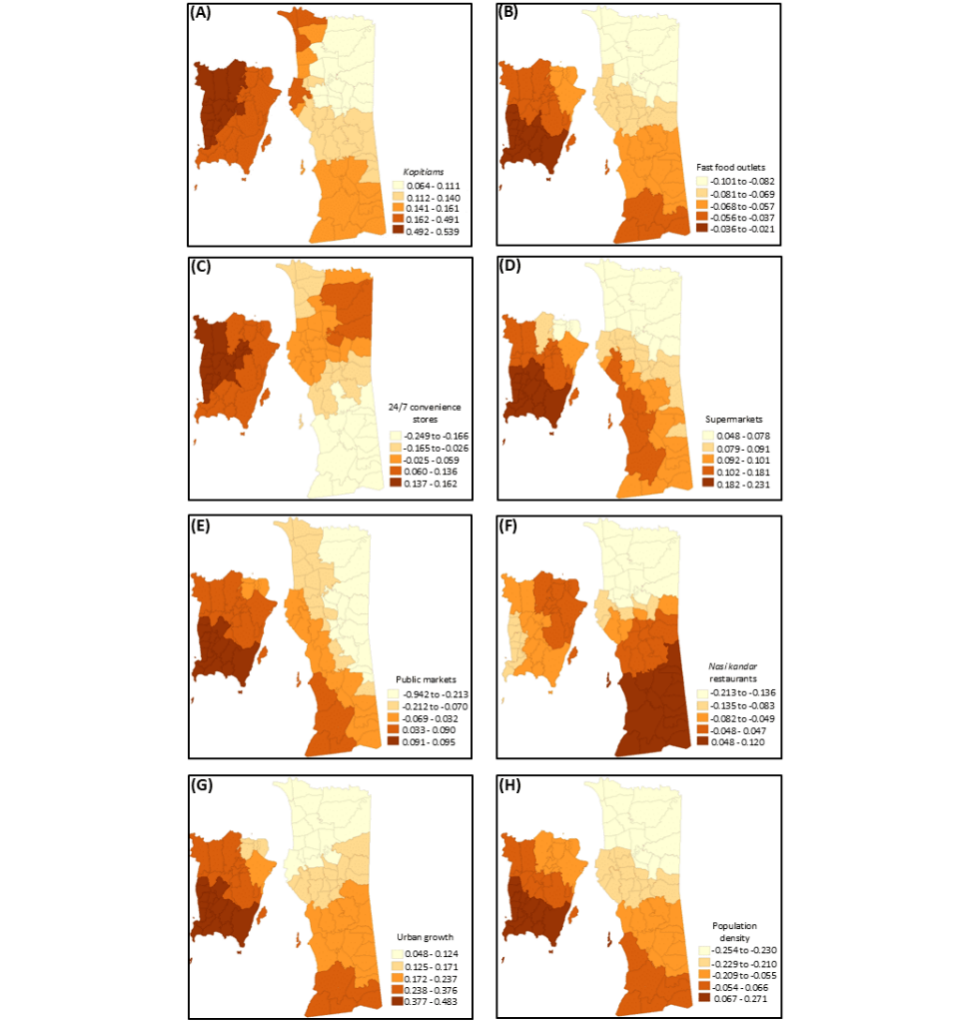
β quantile maps for (A) density of kopitiams per 1000 residents, (B) density of fast food outlets per 1000 residents, (C) density of 24/7 convenience stores per 1000 residents, (D) density of supermarkets per 1000 residents, (E) density of public markets per 1000 residents, (F) density of nasi kandar restaurants per 1000 residents, (G) urban growth rate, and (H) population density.

The β values for the locally adjusted covariates are visualized via quantile maps in Figures 3A-3H. The local MGWR model showed that the density of *kopitiams* per 1000 residents was positively associated with logged T2D rates across *mukims* in almost half of the Barat Daya district (β range 0.492-0.539) followed by *mukims* in the entire Timur Laut district and part of the southern Barat Daya district and some *mukims*, particularly *mukims* 1, 2, 4, and 15, in the Seberang Perai Utara district (β range 0.162-0.491; Figure 3A). In contrast, the density of fast food outlets per 1000 residents was associated with lower logged T2D rates across all *mukims* in Penang, and this negative association was the strongest in almost half of all *mukims* in the Barat Daya district (β range −0.036 to −0.021; Figure 3B).

The density of 24-7 convenience stores per 1000 residents showed the highest associations of logged T2D rates across *mukims* in the west of the Barat Daya district (β range 0.137-0.162) followed by *mukims* in the entire Timur Laut district and the southern Barat Daya district (β range 0.060-0.136). Overall, coefficient estimates ranged from −0.249 to 0.162 with relatively lower logged T2D rates observed across *mukims* within the Seberang Perai Selatan district (Figure 3C). Supermarket density per 1000 residents showed positive relationships with logged T2D rates across all *mukims* in Penang (β range 0.048-0.231); the highest associations with logged T2D rates were found in almost half of the Barat Daya district (β range 0.182-0.231), whereas the lowest associations with logged T2D rates were distributed across *mukims* in the Seberang Perai Utara district (β range 0.048-0.078; Figure 3D).

The density of public markets per 1000 residents was associated with logged T2D rates across *mukims* in almost half of the Barat Daya district (β range 0.091-0.095) followed by *mukims* in the northwest of the Barat Daya district, most *mukims* in the Timur Laut district, and *mukims* in part of the Seberang Perai Selatan district (β range 0.033-0.090); however, the presence of public markets had lower strength of association with logged T2D rates across most *mukims* in the Seberang Perai Utara and Seberang Perai Tengah districts (Figure 3E). Except for *mukims* in the Seberang Perai Selatan district that showed positive relationships between logged T2D rates and the density of *nasi kandar* restaurants per 1000 residents (β range 0.048-0.120), the rest of the *mukims* in Penang showed negative relationships between logged T2D rates and the density of *nasi kandar* restaurants per 1000 residents (Figure 3F).

With respect to urbanization indicators, urban growth was positively associated with logged T2D rates across all *mukims* in Penang, with the highest associations with logged T2D rates being observed across *mukims* in the south of the Barat Daya district (β range 0.377-0.483; Figure 3G). A similar positive association was observed between population density and logged T2D rates, with the highest association with logged T2D rates observed in the south of the Barat Daya district (β range 0.067-0.271), although the rest of the *mukims* across Penang showed negative associations (Figure 3H).

### Estimated Associations Between Density of Food Outlets and Area-Level T2D Cases Within *Mukims* Through Univariate Poisson Regression

Table 3 shows the associations between density of different types of food outlets per 1000 population within *mukims* and area-level T2D cases through univariate Poisson regression. The density of food complexes per 1000 residents (RR=1.63, 95% CI 1.55-1.71; *P*<.001), hawker markets per 1000 residents (RR=1.84, 95% CI 1.68-2.01; *P*<.001), and fast food outlets per 1000 residents (RR=1.14, 95% CI 1.05-1.23; *P*=.002) increased the risk of T2D as compared to the absence of such food outlets. The density of *nasi kandar* restaurants per 1000 residents (RR=0.64, 95% CI 0.61-0.67; *P*<.001), 24-7 convenience stores per 1000 residents (RR=0.80, 95% CI 0.75-0.87; *P*<.001), and supermarkets per 1000 residents (RR=0.81, 95% CI 0.78-0.84; *P*<.001) decreased the risk of T2D as compared to the absence of such food outlets (Table 3).

**Table 3:** The association between density of food outlets and area-level type 2 diabetes cases within mukims through univariate Poisson regression^a^.

Characteristic			Exponentiated Poisson regression coefficient (β crude; 95% CI)		*P* value
**Density of hawker complexes (per 1000 residents)**					.45
	At least one outlet	0.98 (0.94-1.03)			
	No outlet^b^	1			
**Density of food complexes (per 1000 residents)**					<.001^c^
	At least one outlet	1.63 (1.55-1.71)			
	No outlet	1			
**Density of hawker markets (per 1000 residents)**					<.001^c^
	At least one outlet	1.84 (1.68-2.01)			
	No outlet	1			
**Density of *kopitiams* (per 1000 residents)**					.06
	At least one outlet	1.06 (0.99-1.11)			
	No outlet	1			
**Density of *nasi kandar* restaurants (per 1000 residents)**					<.001^c^
	At least one outlet	0.64 (0.61-0.67)			
	No outlet	1			
**Density of fast food outlets (per 1000 residents)**					.002^c^
	At least one outlet	1.14 (1.05-1.23)			
	No outlet	1			
**Density of 24-7 convenience stores (per 1000 residents)**					<.001^c^
	At least one outlet	0.80 (0.75-0.87)			
	No outlet	1			
**Density of public markets (per 1000 residents)**					.15
	At least one outlet	0.97 (0.93-1.01)			
	No outlet	1			
**Density of supermarkets (per 1000 residents)**					<.001^c^
	At least one outlet	0.81 (0.78-0.84)			
	No outlet	1			

^a^β provides the crude rate ratio.

^b^“No outlet” represents the reference category and does not have a 95% CI.

^c^Statistical significance at *P*<.05.

### Estimated Associations Between Density of Food Outlets and Area-Level T2D Cases Within *Mukims* Through Multivariate Poisson Regression Complemented With PAFs

Table 4 shows the association between the density of different types of food outlets per 1000 residents within *mukims* and area-level T2D cases through multivariate Poisson regression complemented with PAFs. For each of the covariates with statistically significant associations, exponentiating the estimated coefficient provided the multiplicative change in the number of T2D cases in the presence of hawker complexes (ARR=0.94, 95% CI 0.89-0.99; *P*=.03), food complexes (ARR=1.24, 95% CI 1.20-1.29; *P*<.001), hawker markets (ARR=1.32, 95% CI 1.22-1.43; *P*<.001), *kopitiams* (ARR=1.09, 95% CI 0.99-1.20; *P*=.08), *nasi kandar* restaurants (ARR=0.63, 95% CI 0.58-0.68; *P*<.001), fast food outlets (ARR=0.92, 95% CI 0.88-0.95; *P*<.001), 24-7 convenience stores (ARR=0.72, 95% CI 0.69-0.75; *P*<.001), public markets (ARR=0.92, 95% CI 0.86-0.97; *P*=.005), and supermarkets (ARR=0.72, 95% CI 0.66-0.78; *P*<.001) per 1000 residents.

**Table 4:** Estimated associations between density of food outlets and area-level type 2 diabetes cases within mukims through multivariate Poisson regression complemented with population-attributable fractions (PAFs)^a^.

Characteristic		Exponentiated Poisson regression coefficient (β adjusted; 95% CI)	*P* value	PAF (%)
**Density of hawker complexes (per 1000 residents)**			.03^c^	−0.07
	At least one outlet	0.94 (0.89-0.99)		
	No outlet	1^b^		
**Density of food complexes (per 1000 residents)**			<.001^c^	0.21
	At least one outlet	1.24 (1.20-1.29)		
	No outlet	1		
**Density of hawker markets (per 1000 residents)**			<.001^c^	0.27
	At least one outlet	1.32 (1.22-1.43)		
	No outlet	1		
**Density of** ***kopitiams*** **(per 1000 residents)**			.08	0.09
	At least one outlet	1.09 (0.99-1.20)		
	No outlet	1		
**Density of** ***nasi kandar*** **restaurants (per 1000 residents)**			<.001^c^	−0.64
	At least one outlet	0.63 (0.58-0.68)		
	No outlet	1		
**Density of fast food outlets (per 1000 residents)**			<.001^c^	−0.10
	At least one outlet	0.92 (0.88-0.95)		
	No outlet	1		
**Density of 24-7 convenience stores (per 1000 residents)**			<.001^c^	−0.43
	At least one outlet	0.72 (0.69-0.75)		
	No outlet	1		
**Density of public markets (per 1000 residents)**			.005^c^	−0.10
	At least one outlet	0.92 (0.86-0.97)		
	No outlet	1		
**Density of supermarkets (per 1000 residents)**			<.001^c^	−0.43
	At least one outlet	0.72 (0.66-0.78)		
	No outlet	1		

^a^β provides the adjusted rate ratio.

^b^“No outlet” represents the reference category and does not have a 95% CI.

^c^Statistical significance at *P*<.05.

With regard to parameter interpretations of PAFs, it was found that, if local neighborhoods (within *mukims*) underwent township restructuring to remove food complexes, hawker markets, or *kopitiams*, the result would be an estimated reduction of 0.21%, 0.27%, and 0.09% in the number of *mukim*-level T2D cases, respectively. In contrast, if the local neighborhoods (within *mukims*) underwent township restructuring to add hawker complexes, *nasi kandar* restaurants, fast food outlets, 24-7 convenience stores, public markets or supermarkets, the result would be an estimated reduction of 0.07% to 0.64% in the number of residents within those *mukims* having T2D (Table 4).

## Discussion

### Principal Findings

Our aim was to first assess how close commercial food establishments are to the residences of individuals with T2D within neighborhoods; subsequently, explore how rates of T2D differ by location; and, finally, evaluate the extent to which the density of specific food outlets in a neighborhood needs to be modified to manage local T2D rates. Our analysis revealed variations in the associations between proximity to food outlets and the BMI of T2D cases when different food outlets were populated in the hierarchical model adjusted for age, with model performance ranging from 1.1% to 6.5%. The MGWR β coefficients showed how different food outlet density varied by space, indicating areas that should be prioritized for local public health interventions. Finally, the PAFs suggested that, if local neighborhoods underwent township restructuring to remove food complexes, hawker markets, or *kopitiams*, an estimated reduction of 0.21%, 0.27%, and 0.09%, respectively, in the risk of T2D cases in Penang would be anticipated but, if local neighborhoods underwent township restructuring to add hawker complexes, *nasi kandar* restaurants, fast food outlets, 24-7 convenience stores, public markets or supermarkets, an estimated reduction of between 0.07% and 0.64% in the number of residents with risk of T2D was estimated. These findings provide a better overview of a local neighborhood landscape regarding where interventions should be focused to tackle local T2D burden.

### Comparison With Prior Work

Our linear regression approach assisted us in understanding how much variation persists and how food environments change with neighborhood development and urbanization processes. In the first step, if the neighborhood was only structured with access to hawker food outlets, the likely change in the BMI of T2D cases would account for approximately 1.1% of the variance in the model, whereas in the subsequent step, if the town was further structured or developed to facilitate accessibility to *kopitiams*, *nasi kandar* restaurants, and fast food outlets, the change in BMI among T2D cases was likely to account for approximately 1.4% of the variance. If the town became more populated with convenience stores, public markets, and supermarkets for access by local communities, the likely change in BMI would account for approximately 1.5% of the variance. Finally, to make it more sensible as towns are inhabited by individuals of different demographics, the final model that adjusted for age yielded a huge increase in the BMI change among T2D cases, accounting for 6.5% of the overall variance. These findings highlight that proximity to food outlets was associated with BMI concurrently mediated by age [40-43], perhaps based on different food preferences and ease of access to neighborhood food outlets as perceived by different age groups.

In the final model, proximity to food complexes and hawker markets within a kilometer showed negative associations with BMI, whereas proximity to *kopitiams* within 1 km showed positive associations with BMI. These local eateries offer different food choices and are common venues for local communities to access for breakfast, lunch, or dinner [44]. Similarly, while proximity to *kopitiams* was positively associated with BMI change in T2D cases, the direction of the association was negative for food complexes and hawker markets, likely attributed to the local food classifications or subject to the effect of the spatial scale applied for this investigation [45].

Proximity to fast food outlets showed negative associations with BMI, consistent with the findings reported by Jeffery et al [46] but inconsistent with those reported by Kusuma et al [11] and van Erpecum et al [28]. The negative association between fast food outlet proximity and BMI mirrors some earlier results [47]; however, it is important to consider the ecologic nature of our estimates and the small local sample sizes involved in estimating local impacts of fast food access [48]. Supermarket proximity showed no statistically significant relationship with BMI change, inconsistent with the work by Garg et al [49]. Proximity to 24-7 convenience stores and BMI change showed negative associations, with the literature showing variations across different geographical areas [50,51]. Proximity to public markets unexpectedly showed positive associations with BMI change; however, such a finding was also anticipated in a previous study that suggested that low numbers of public markets in different areas could be associated with a nonprotective effect of public market accessibility on BMI change among individuals with T2D [52].

The local bandwidths of the MGWR model showed that associations among local T2D prevalence rates; the density of fast food outlets, supermarkets, public markets, and *nasi kandar* restaurants; and urban growth occurred at a global scale, whereas the association with the density of *kopitiams* and 24-7 convenience stores occurred at a regional scale and, finally, associations between T2D prevalence and population density occurred at the local scale. The adjusted *R*^2^ quantile maps and β weight quantile maps from the MGWR regression could provide clues for priority setting [35,53]. This study found higher local *R*^2^ values from the pooled covariates in the MGWR model that ranged from 0.53 to 0.62 covering all *mukims* in the Timur Laut district and half of all *mukims* in the Barat Daya district. These areas are urbanized towns with higher population density nested within neighborhoods with high densities of food establishments offering food deemed to be *unhealthy*. The overall adjusted *R*^2^ values ranged from 0.15 to 0.62, with lower values observed across *mukims* in mainland Penang, particularly in Seberang Perai Selatan and parts of *mukims* in Seberang Perai Tengah and Seberang Perai Utara. These areas show lower urban growth and population density, with food establishments operating according to community needs or demands. The β weight coefficient maps obtained from the MGWR results support the trend of highly concentrated *kopitiams* and 24-7 convenience stores increasing T2D rates in almost half of the *mukims* in the Barat Daya district, whereas high concentrations of supermarkets and public markets in parts of *mukims* across the Barat Daya and Timur Laut districts showed positive correlations with T2D rates alongside increased urban growth and population density as compared to the more rural areas in the mainland. Examining relationships across diverse geographical settings is a complex endeavor, as in this study, where areal urbanization processes vary across the most urbanized towns to suburban or small towns and, finally, rural settings, where patterns of food establishments and their correlation with T2D could vary substantially [54].

Previous works have identified supermarkets as having more diverse and healthy food options as compared to convenience stores; however, the availability of the latter in rural settings or areas of lower urban growth as the main source of food purchases may increase local communities’ risk of T2D [55,56]. Nevertheless, fast food outlet concentrations in those areas showed negative correlations with T2D rates, and the plausibility of such associations could be attributed to classifications of definitions from the available sources [7]. Except for *mukims* in the Seberang Perai Selatan district that showed positive relationships between T2D rates and the density of *nasi kandar* restaurants, the rest of the areas in Penang showed negative relationships. The results of the MGWR analysis were in general alignment with the global model; however, the local insights of the MGWR estimates pose questions as to where significant associations occur, the local strength of such associations, and how these could be prioritized for local interventions. A similar argument was put forward in a previous study that used MGWR [35].

Our work illustrates how the neighborhood food environment influences T2D rates through local patterning. Traditionally, health care sector prevention efforts have frequently directed interventions toward diet, weight loss, or lifestyle [12,57], but these interventions are difficult and a costly enterprise to upscale to population-level interventions [58]. Despite these challenges, policy advocates and decision makers are aware of the need for coordinated multisectoral prevention action to promote health within neighborhoods throughout the human life course. Unifying multiple stakeholders or decision makers requires a single convincing metric to initiate preventive actions in what our results show is a complicated system with multiple moving parts. One metric considered in this work was PAFs [45,59], allowing for the epidemiological interpretation that if local neighborhoods underwent township restructuring to remove food complexes, hawker markets, or *kopitiams*, an estimated reduction of 0.21%, 0.27%, and 0.09%, respectively, in the number of T2D cases would be anticipated. Similarly, if local neighborhoods underwent township restructuring to add hawker complexes, *nasi kandar* restaurants, fast food outlets, 24-7 convenience stores, public markets or supermarkets, an estimated reduction of 0.07% to 0.64% in the number of residents with T2D was estimated. While the former was convincing, the latter could be debatable because of the local classification of those food establishments [7] and the spatial scale that was used [45].

### Policy Implications

Our 3 focused objectives yielded 3 crucial metrics that set the foundation for public health policymaking in the quest to reduce local T2D burden in Penang. First, our hierarchical multivariable linear regression showed how the distance of different food outlets from the locus of analysis was associated with change in the BMI of T2D cases in Penang. We documented the strength of association and model performance when a particular food outlet was added in a hierarchical way to the model adjusted for age—keeping in mind how neighborhoods undergo restructuring in line with urbanization processes—thus allowing for interpretations regarding dietary choices and accessibility among different age groups. Proximity measures may influence zoning decisions by local authorities to facilitate food outlet licensing within neighborhoods while creating opportunities to advocate for healthier food options.

Second, across neighborhoods, the area-level local MGWR β weights and their residuals and bandwidths revealed how the factors pooled together were associated with the concentration of T2D rates by areal *mukims* and, if interventions or policies were planned to be executed, whether they should be targeted at one specific focal area or be applied to the entire population in that area. For example, if the density of certain food outlets were found to be associated significantly with area-level T2D rates across *mukims*, local city councils, local assemblypersons, township neighborhood planners, or local community boards could advocate for the relocation or removal of those food outlets, limit license approval for newer establishments of similar food outlets that could have detrimental effects on health, or even plan community health education programs on healthy food purchase. This approach could minimize the harmful effects of commercial determinants of health such as unhealthy food promotion or availability to neighborhood residents.

The MGWR bandwidth metrics convey important messages for public health responses, providing evidence regarding at what scale and for what targets local programs or interventions should be implemented based on their predicted effect magnitude, as well as providing insight into how interventions should be planned, implemented, and evaluated. We recommend an interplay between health promotion (ie, how local health departments, community organizations, and policy makers could foster the overall well-being of communities by addressing the commercial determinants of food environments, such as restructuring the town) and health education (ie, how community health leaders, health care providers, or government agencies could impart knowledge or skills to promote healthy behaviors, such as community programs on healthy food purchase or implementation of local subsidized voucher programs for purchasing healthy food options) interventions [60]. The former would be more appropriate to tackle associations at a global or regional scale, whereas the latter would be more appropriate for a local scale.

Alongside the synthesis of MGWR β weights and bandwidths, the local *R*^2^ values could assist public health policy makers in understanding where certain interventions (eg, food outlet relocation, township restructuring, or targeted community-based behavioral interventions) need to be prioritized. When the model’s predictive power is higher across specific regions or areas, intervening in local factors with associated high associations and in areas exhibiting high local *R*-squared values, it would be wise for additional investigations to be conducted first to uncover other potential risk factors before interventions are implemented for optimal outcomes that provide value for money.

While the RRs estimated changes in the local T2D burden in conjunction with the local measures of food outlet density within a neighborhood, the PAFs provide another important interpretable metric for policy makers for food environment interventions; that is, if local neighborhoods (within *mukims*) undergo township restructuring to remove certain food premises, what would be the estimated reduction in T2D cases within those *mukims.* These metrics complement the findings from the MGWR (ie, measures across *mukims* or their neighbors), but in this case, PAFs are interpretable within *mukims* (ie, without their neighbors), which motivates a more holistic approach useful for policy planning in developing or restructuring towns to include healthy urbanism neighborhood features.

### Limitations

We note that our interpretations are not causal but associative (ie, using ecological and cross-sectional designs); however, these designs are relatively suitable to frame our regional findings for policy implications. We opted for an ecological study design that can be subject to ecological fallacy (ie, interpretation of risk factors observed at the group level that cannot be individualized). While this study acknowledged this limitation by convention, it should be considered from a geographical point of view by interpreting where and why T2D occurs as the aim of any spatial epidemiological study is to provide evidence by place for targeted interventions. We frame our results regionally as many public health policies are usually made at the aggregate level involving populations or communities within neighborhoods.

We note that MGWR is a regression model that assumes that the residuals follow a normal distribution. To see whether we had independent observations that violated Tobler’s First Law of Geography, we fit a standard linear ordinary least squares regression with the log-rates as outcome variables using independent, normally distributed error terms and compared model fit to guard against this potential limitation. As an alternative, we propose to fit a Poisson regression with spatially correlated random effects or the integrated nested Laplace approximation for similar data analysis in the future.

Next, we acknowledge that, while addresses provide point locations for residences and food outlets, we are required to preserve patient confidentiality and anonymity. One limitation of our analysis is that it currently does not allow each individual’s mobility and preferences regarding food purchase to result in dining patterns covering a broader area than the closest food outlets. Although our work focused on small-area summaries of both our outcome and the exposure to nearby food outlets, our results can motivate future research to explore finer-scale associations between point patterns of residence and mobility zones using point process summaries such as kernel density estimates [25].

In addition, although there is evidence suggesting that ethnicity plays a critical role in determining associations with T2D at an individual level, this scenario may not result in the same associations with risk measured at an area level as different proportions of ethnic groups (ie, either ethnic minority or majority groups) may reside across different settings. Under these circumstances, we did not include ethnic proportions by areas as a potential variable to be adjusted for in any models as this is not a local feature that can be changed via policies. Future work can adopt a more nuanced approach by exploring interactions between local policy impacts and local analysis interpretations stratified by ethnicity.

Finally, while we established the correlation between T2D rates and the density of food outlets by administrative levels, we caution that our approach and analyses may be subject to the modifiable areal unit problem (ie, a geographic manifestation of the ecological fallacy where estimated associations may differ when translated to smaller areal units, such as local authority areas, or larger areal units, such as administrative districts) when it is replicated within different administrative boundaries [13]. We also note that, based on the establishment type and the food choices being offered, the estimates for PAFs were relatively low for hawker complexes and fast food outlets. Under modern commercialization landscapes, it would be difficult to eliminate such establishments completely; however, we could first reduce their density of availability, which could result in reduced health impacts on local neighborhoods. Another relevant query is to find what spatial extent of the densities of food outlets is relevant to dietary behaviors that, in turn, could accelerate the risk of T2D, a scenario known as the uncertain geographic context problem [61] that is difficult to adjust for in spatial studies.

### Conclusions

We derived 3 important indicators to engage local epidemiologists, township planners, and public health policy advocates in setting priorities for T2D control in Penang. Our results showed how BMI and age change with different proximities to food outlets among residents with T2D, followed by how the density of different food outlets in different spaces was correlated at different levels with T2D rates across space in Penang. We next explored how much the town’s neighborhood food environment should be restructured to reduce the incidence of T2D in Penang. Our findings suggest that areas with high T2D rates and different food outlet densities vary by *mukim* and coupled with the relative ease of access adjusting for age should be considered for targeted interventions.

Using spatial analytics in epidemiological investigations and studies has offered better alternatives for researchers and public health advocates to explore real-time local disease burden within communities across areas. This approach can explore distal determinants of health as compared to proximal ones to identify local area-level risks for targeted intervention. We note that T2D is a common problem, but despite efforts to control and interventions to limit the risk of the disease, T2D seems to be increasing across the population. Although interventions have been targeted to increase awareness of the risk or promote healthy lifestyle behaviors at the individual level, public health systems continue to struggle in tackling the T2D burden and its complications. Our work offers insights into distal determinants of health within real-time neighborhood features across the urbanization process and a paradigm shift toward crafting strategies at the area level.

## Appendix

Multimedia Appendix 1 Malaysian Standard Industrial Classification codes for food, beverages, household goods, and grocery services.

Multimedia Appendix 2 Locally estimated scatterplot smoothing regression interpolation on influence of proximity of hawker complexes, food complexes and hawker markets on the BMI of type 2 diabetes cases.

Multimedia Appendix 3 Locally estimated scatterplot smoothing regression interpolation on influence of proximity of *nasi kandar*, *kopitiam* and fast-food outlets on the BMI of type 2 diabetes cases.

Multimedia Appendix 4 Locally estimated scatterplot smoothing regression interpolation on influence of proximity of 24-7 convenience stores, public markets and supermarkets on the BMI of type 2 diabetes cases.

Multimedia Appendix 5 Multiscale geographically weighted regression results and model performance.

## Abbreviations

ARR: adjusted rate ratio
ENP: effective number of parameters
km: kilometers
LOESS: locally estimated scatterplot smoothing
MGWR: multiscale geographically weighted regression
NDR: National Diabetes Registry
PAF: population attributable fraction
RR: rate ratio
T2D: type 2 diabetes

## Data Availability

The datasets generated or analyzed during this study are available from the corresponding author on reasonable request.
