# Supplementary material for: Impact of Commercial Food Environments on Local Type 2 Diabetes Burden: Cross-Sectional and Ecological Multimodeling Study
Source: JMIR Public Health Surveill. 2025 Sep 8;11:e70045. doi: 10.2196/70045 (PMC12455153; doi:10.2196/70045)
Supplement: Multimedia Appendix 1 [file publichealth_v11i1e70045_app1.docx]

Malaysian Standard Industrial Classification Codes for Food, Beverages, Household Goods, and Grocery Services (MSIC 2008)

| **MSIC Code** | **Division** | **Group & Class** | **Description** | **Local Classification** | **Outlets Type** |
| --- | --- | --- | --- | --- | --- |
| 56106 | Food and beverage service activities | Restaurant and mobile food service activities | Food stalls/hawkers | Hawker complexes (hawker stalls located outdoor without air-conditioning service) | Outdoor hawker stalls within food courts, complexes, pavilion of tastes, food bazaars, and long beach food halls. |
|  |  |  |  | Food complexes (hawker stalls located indoor with air-conditioning service) | Indoor hawker stalls within food complexes, food bazaars, and food houses. |
| 56107 | Food and beverage service activities | Restaurant and mobile food service activities | Food or beverage, food and beverage preparation in market stalls/hawkers (include market/ stall/ hawkers at *pasar tani,* and night market) | Hawker markets | Hawker stalls within market annexes, *pasar tani*, morning market (*pasar pagi*), night market (*pasar malam*), uptown, midtown, and temporary hawker lots (TPS). |
|  |  |  |  | Public markets (primarily sells fruits, vegetables, fish, meat, etc.) | Local neighborhood public general markets. |

| 56302 | Food and beverage service activities | Beverage serving activities | Coffee shops | Kopitiams (venue where local or franchise coffee beverages or baked products could be purchased) | Franchise outlets (e.g., Coffee Bean, St. Presso Coffee, Starbucks, Old Town White Coffee); Espresso bar (e.g., KAFFA Espresso Bar); Donuts & Coffee (e.g., J. Co Donuts & Coffee, Big Apple Donuts & Coffee); Heritage coffee (e.g., D’ Heritage Coffee & Chocolate); Cafe (e.g., Dome Cafe, Coffee Time Cafe); Bread & Coffee (e.g., Bread King and Coffee); Coffee Roastery (e.g., Frank Laurent Coffee Roasters); Local favorites & classics (e.g., Homestyle Kopitiam, Georgetown White Coffee, Kopitan Classic, Pinang Kopitiam, Roti Bakar Kopitiam, Station Kopitiam). |
| --- | --- | --- | --- | --- | --- |
| 56101 | Food and beverage service activities | Restaurant and mobile food service activities | Restaurants and restaurant cum night clubs | *Nasi kandar* restaurants (seated venue where local food delicacy is purchased and primarily eaten on site) | Local chain of *nasi kandar* restaurants. |
| 56103 | Food and beverage service activities | Restaurant and mobile food service activities | Fast-food restaurants | Fast-food restaurants | A&W, Auntie Anne’s, Ayamas, Baskin Robbins, Burger King, Dave’s Deli, Domino’s Pizza, Haagen Dazs, Kenny Rogers Roasters, KFC, Marry Brown, McDonald’s, Nando’s, Pappa Rich, Pizza Hut, Rasamas, Roti Boy, Secret Recipe, Subway, Sushi King, Swensen’s, Texas Chicken, The Chicken Rice Shop, The Manhattan Fish Market, Wendy’s. |
| 47114 | Retail trade, except of motor vehicles and motorcycles | Retail sale in non-specialized stores | Retail sale in non-specialized stores with food, beverages or tobacco predominating | 24/7 Convenience stores | 24 Xpress, 7-Eleven, 8 Pagi, 98 Mart, Azira Mart, D-Lima, Happy Mart, Juara Mart, Nasmir Mart, Puz Mart. |
| 47112 | Retail trade, except of motor vehicles and motorcycles | Retail sale in non-specialized stores | Retail sale in non-specialized stores with food, beverages or tobacco predominating | Supermarkets | AEON Supermarket, Bandar Baru Supermarket, Billion Mart, C-Mart, Cold Storage, Econsave, GAMA Supermarket, Giant Supermarket, Mydin, Pacific, Parkson Supermarket, Pasaraya 800, SAM’S Groceria, Sunshine, Tesco, Yawata. |
