# Supplementary material for: Impact of Commercial Food Environments on Local Type 2 Diabetes Burden: Cross-Sectional and Ecological Multimodeling Study
Source: JMIR Public Health Surveill. 2025 Sep 8;11:e70045. doi: 10.2196/70045 (PMC12455153; doi:10.2196/70045)

**Locally estimated scatterplot smoothing regression interpolation on influence of proximity of hawker complexes, food complexes and hawker markets on the BMI of type 2 diabetes cases**

Figure displays the locally estimated scatterplot smoothing (LOESS) regression interpolation on the distance influence of hawker complexes, food complexes and hawker markets with body mass index (BMI) of diabetes cases in Penang (n=11047). The associations between BMI and all hawker outlets showed a relatively smooth fit line, indicative of linear correlations, thus being subjected to linear regressions in the next steps.


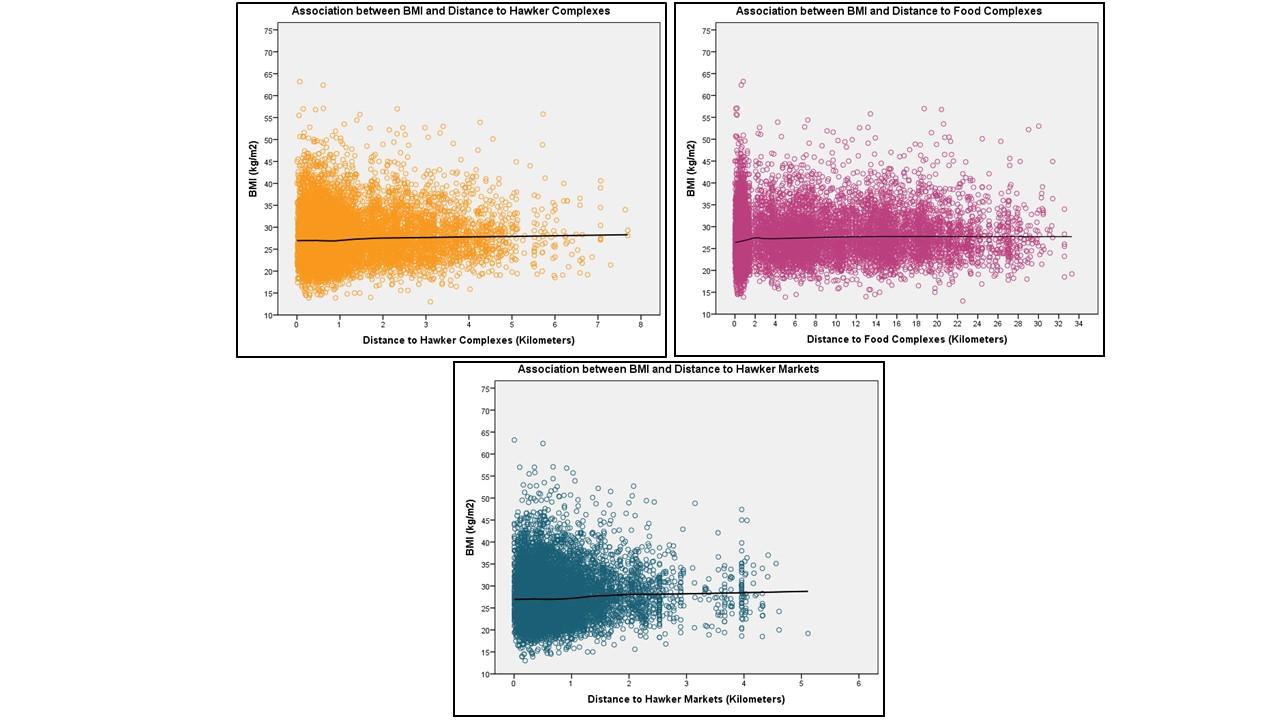

Supplement: Multimedia Appendix 2 [file publichealth_v11i1e70045_app2.docx]
