# Supplementary material for: Impact of Commercial Food Environments on Local Type 2 Diabetes Burden: Cross-Sectional and Ecological Multimodeling Study
Source: JMIR Public Health Surveill. 2025 Sep 8;11:e70045. doi: 10.2196/70045 (PMC12455153; doi:10.2196/70045)

**Locally estimated scatterplot smoothing regression interpolation on influence of proximity of 24/7 convenience stores*,* public markets and supermarkets on the BMI of type 2 diabetes cases**

Figure displays the locally estimated scatterplot smoothing (LOESS) regression interpolation on the distance influence of 24/7 convenience stores, public markets and supermarkets with body mass index (BMI) of diabetes cases in Penang (n=11047). The associations between BMI and all food outlets showed a relatively smooth fit line, indicative of linear correlations, thus being subjected to linear regressions in the next steps.


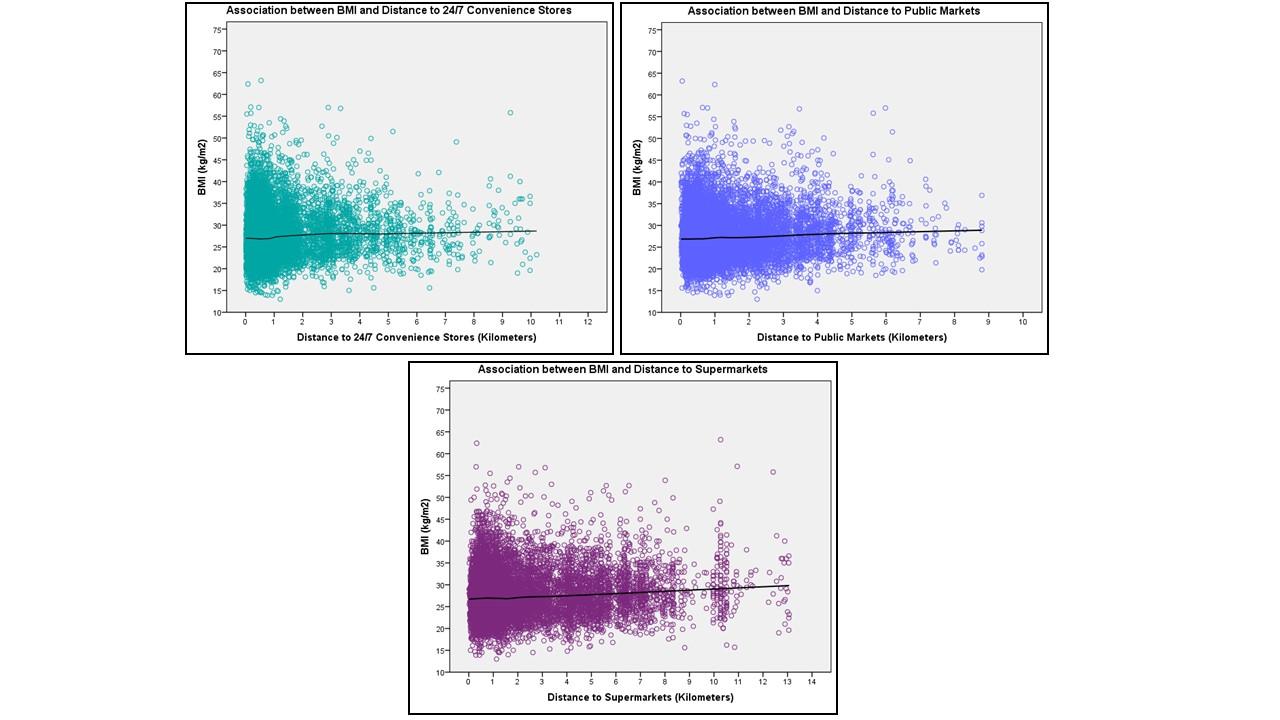

Supplement: Multimedia Appendix 4 [file publichealth_v11i1e70045_app4.docx]
