# Supplementary material for: Impact of Commercial Food Environments on Local Type 2 Diabetes Burden: Cross-Sectional and Ecological Multimodeling Study
Source: JMIR Public Health Surveill. 2025 Sep 8;11:e70045. doi: 10.2196/70045 (PMC12455153; doi:10.2196/70045)
Supplement: Multimedia Appendix 5 [file publichealth_v11i1e70045_app5.docx]

### **Multiscale GWR Results and Model Performance**

The Akaike Information Criterion (AIC) for MGWR was 207.85, reduced from that for the global OLS regression model (244.41), suggesting that the local MGWR provided a better fit to data. The MGWR model, a local model accounted for 53.8% of the total variance in local logged T2D rates, after adjusting for local densities of food outlets and urban indicators (refer table below).

The bandwidths pertaining to each explanatory covariate included in the MGWR model are listed in the table below, with comparisons made to a single bandwidth of 83 nearest neighbours. Five associations were found to occur at an effectively global scale (i.e., density of fast-food outlets per 1000 residents, density of supermarkets per 1000 residents, density of public markets per 1000 residents, density of *nasi kandar* restaurants per 1000 residents, and urban growth rate) with bandwidths indicating nearly all data being included in the local subset of the modelled covariates; two processes were seen to have occurred at a regional scale (density of *kopitiams* per 1000 residents and density of 24/7 convenience stores per 1000 residents) with lower bandwidth values than the global scale of nearest neighbours; and another one process perceived to vary locally (i.e., population density), yielding relatively smaller bandwidths than the regional scale (refer table below).

The effective number of parameters showed similar consistencies for interpretations of spatial heterogeneity; larger ENP values occurred relative to smaller bandwidths suggesting greater spatial heterogeneity, while the bandwidths approaching to the total number of observations (i.e., spatial units) with ENP values nearing 1 indicated little spatial variations in establishing the strength of associations. Covariate-specific adjusted *t*-values identified *mukims* with statistically significant relationships between the density of *kopitiams* per 1000 residents (adjusted *t*-value=2.316), density of fast food outlets per 1000 residents (adjusted *t-*value=2.111), density of 24/7 convenience stores per 1000 residents (adjusted *t*-value=2.371), density of supermarkets per 1000 residents (adjusted *t*-value=2.101), density of public markets per 1000 residents (adjusted *t*-value = 2.031), density of *nasi kandar* restaurants per 1000 residents (adjusted *t*-value = 2.189), urban growth rate (adjusted *t*-value=2.198), population density (adjusted *t*-value=2.256) and logged T2D rates (refer table below). The degree of freedom (df) was 65.835.

**Multiscale GWR model on the association between neighbourhood food environment, urban indicators, and logged T2D rates by *mukims* in Pulau Pinang**

| **Indicators** | **OLS Model** | | | **MGWR Model** | | | |
| --- | --- | --- | --- | --- | --- | --- | --- |
|  | ***ß*** | **SE** | ***P*-value** | ***ß* (Mean)** | **Bandwidth (95% BCI)** | **ENP** | **Adjusted *t*-value (95%)** |
| Density of *kopitiams* (per 1000 residents) | 0.225 | 0.136 | .098 | 0.256 | 60.0 (52.0, 67.0) | 2.170 | 2.316 |
| Density of fast food outlets (per 1000 residents) | -0.009 | 0.147 | .950 | - 0.061 | 81.0 (58.8, 81.0) | 1.322 | 2.111 |
| Density of 24/7 convenience stores (per 1000 residents) | -0.040 | 0.130 | .758 | 0.028 | 58.0 (52.0, 67.0) | 2.488 | 2.371 |
| Density of supermarkets (per 1000 residents) | 0.067 | 0.139 | .629 | 0.122 | 75.0 (58.0, 77.0) | 1.291 | 2.101 |
| Density of public markets (per 1000 residents) | 0.027 | 0.111 | .804 | 0.067 | 73.0 (58.0, 77.0) | 1.100 | 2.031 |
| Density of *nasi kandar* restaurants (per 1000 residents) | 0.024 | 0.141 | .864 | - 0.064 | 81.0 (58.0, 81.0) | 1.591 | 2.189 |
| Urban growth rate (%) | 0.197 | 0.112 | .079 | 0.189 | 75.0 (58.0, 77.0) | 1.623 | 2.198 |
| Population density (inhabitants/km^2^) | 0.091 | 0.113 | .424 | 0.080 | 46.0 (44.0, 67.0) | 1.869 | 2.256 |
| **Model Performance** | | | | | | | |
| Number of observations | 83 | | | 83 | | | |
| Akaike Information Criterion (AIC) | 244.41 | | | 207.85 | | | |
| R-squared | 0.104 | | | 0.538 | | | |

***Note:*** *(1) The OLS model was yielded by default prior to the extension of an MGWR model; (2) ENP denotes Effective Number of Parameters; (3) Local regressions of MGWR does not govern p-values because the beta values change over space; (4) Adjustment of spatial variation in associations is important at this scale.*
